# Phenotypic and Genotypic Antibiotic Resistance Patterns in *Helicobacter pylori* Strains From Ethnically Diverse Population in México

**DOI:** 10.3389/fcimb.2020.539115

**Published:** 2021-02-11

**Authors:** Margarita Camorlinga-Ponce, Alejandro Gómez-Delgado, Emmanuel Aguilar-Zamora, Roberto C. Torres, Silvia Giono-Cerezo, Antonio Escobar-Ogaz, Javier Torres

**Affiliations:** ^1^Unidad de Investigación en Enfermedades Infecciosas, UMAE Pediatría, Instituto Mexicano del Seguro Social, Ciudad de México, Mexico; ^2^Posgrado en Biomedicina y Biotecnología Molecular, Escuela Nacional de Ciencias Biológicas, Instituto Politécnico Nacional, Ciudad de México, Mexico; ^3^Departamento de Microbiología, Escuela Nacional de Ciencias Biológicas, Instituto Politécnico Nacional, Ciudad de México, Mexico

**Keywords:** *Helicobacter pylori*, antibiotic resistance, whole genome sequencing, phenotype, genotype, indigenous communities

## Abstract

*Helicobacter pylori* strains carry a range of mutations in genes that confer antimicrobial resistance and restrict the available options to treat the infection. Latin America is a region that conserve a large number of indigenous communities relatively isolated that practice a traditional medicine without consumption of drugs. We hypothesized that rates of antibiotic resistance are lower in these communities. Recent progress in whole-genome sequencing has allowed the study of drug susceptibility by searching for the known mutations associated with antibiotic resistance. The aim of this work was to study trends of antibiotic resistance over a 20-year period in Mexican *H. pylori* strains and to compare susceptibility between strains from Mexican mestizos and from indigenous population; we also aimed to learn the prevalence of mutational patterns in genes *gyrA, gyrB, rdxA*, *frxA, rpsU, omp11*, *dppA*, and 23S rRNA and its association with phenotypic tests. Resistance to clarithromycin, metronidazole, amoxicillin and levofloxacin was determined in167 *H. pylori* isolates by E-test, and the occurrence of mutational patterns in specific genes was determined by whole genome sequencing (WGS). The trend of resistance over 20 years in mestizo isolates showed significant resistant increase for clarithromycin and levofloxacin to frequencies that banned its clinical use. Resistance in *H. pylori* isolates of native communities was lower for all antibiotics tested. Phenotypic resistance showed good to moderate correlation with genotypic tests. Genetic methods for characterizing antibiotic resistance require further validation in each population.

## Introduction

Drug resistance is recognized as one of the major threats to worldwide public health (Laxminarayan et al., 2013). The problem is a highly pressing issue because of the rapid spread of multi-drug resistant bacteria, some of which are no longer treatable with the available antibiotics (Dadgostar, 2019). Antibiotic resistance is the main risk factor for failure to respond to treatment of *H. pylori* infection (Alfizah et al., 2014)*. H. pylori* is an ancient microorganism that has co-evolved with man for at least 60,000 years (Moodley et al., 2012); still, it represents a significant public health issue since it is the main risk factor for gastric cancer, which is the third cause of death because of all cancers (Hooi et al., 2017). It is estimated that over 90% of all cases of stomach cancer are attributed to *H. pylori* (Moss, 2017). Eradication treatment against *H. pylori* may halt the progression of chronic atrophic gastritis and intestinal metaplasia, preventing the development of gastric cancer (Mera et al., 2005; Fuccio et al., 2009).

*H. pylori* is a small, curved, motile, Gram-negative bacillus that colonizes the stomach mucosa of more than 50% of the world population, particularly in developing countries of Africa, Latin America, the Caribbean and Asia (Sjomina et al., 2018). In contrast, the lowest prevalence is reported in Northern America and Oceania, whereas it is declining in Japan (Leja et al., 2016).

Although there is no global recommendation on the regimen for eradication of *H. pylori*, there are regional consensus (e.g., European, Latin American, Asiatic) (Rollan et al., 2014; Malfertheiner et al., 2017; Sheu et al., 2017) that in the last decades have recommended mostly a “standard” triple therapy (proton pump inhibitor + clarithromycin + amoxicillin) as the first choice of treatment. However, during the last years, most of these regions have reconsidered their recommendations, because of the increase in resistance to clarithromycin (Thung et al., 2016). Current recommendations vary among regions because susceptibility to drugs is different in each geographic area, but also because of other factors like availability and cost of the drugs. The most recently recommended drugs for *H. pylori* eradication include fluoroquinolones and rifamycin (Goderska et al., 2018).

The *H. pylori* strains may carry a range of mutations in genes that confer antimicrobial resistance and limit the available options to treat *H. pylori* infection. Resistance to clarithromycin is commonly associated to three point mutations in the 23S ribosomal RNA (rRNA) gene, A2143G (69.8%), A2142G (11.7%), and A2142C (2.6%) (Thung et al., 2016); whereas other less frequent mutations include A2115G, G2141A, A2144T, and T2289C. Mutations C2694A and T2717C have been associated with low resistance levels (Marques et al., 2020). Other point mutations have been described in the literature (A1821G, G1826A, and T1830C, C2245T, G2224A, but their role in clarithromycin resistance requires confirmation (Rimbara et al., 2007; Alarcón-Millán et al., 2016; Hashemi et al., 2019). These mutations prevent the interaction of the macrolide with the 23S rRNA. The prevalence of *H. pylori* resistance to clarithromycin varies among regions. Reported resistance in West/central and South Europe is >20%, and in North Europe it is <10% (Megraud et al., 2013); whereas in strains from Latin American countries the overall prevalence of resistance is 12%, although frequencies ranged from 0% to 60% (Camargo et al., 2015).

Reported *H. pylori* resistance to metronidazole is 33.1% in Europe and 40% in the USA, whereas higher rates have been reported in developing countries, with frequencies as high as 80% (Rollan et al., 2014; Thung et al., 2016); still, metronidazole is frequently used in eradication regimens (Fiorini et al., 2018). Mutations in the *rdxA* gene have been identified as the main cause of resistance to this drug (Goodwin et al., 1998). Although it has been found that mutations in other genes could also influence resistance to metronidazole, including *frxA*, *mdaB, omp11, ddpA or rpsU* genes, and genes involved in transcription regulation of *rdxA* or in overexpression of *hfA* efflux pump; although additional studies are required to clearly demonstrate their association with metronidazole resistance (Alba et al., 2017; Saranathan et al., 2020). *H. pylori* resistance to amoxicillin has remained low worldwide, with reported frequencies of <2% in European countries (Thung et al., 2016) and 6.6% in Bangladesh (Nishizawa and Suzuki, 2014). Amoxicillin binds to penicillin binding proteins (PBP) interfering with peptidoglycan synthesis causing lysis of replicating bacteria and resistance is mainly associated with mutations in the *pbp* genes (Nishizawa and Suzuki, 2014). Resistance to fluoroquinolones has been determined in a few countries, with rates of 3.3% in France, 34.5% in China, 31.9% in the USA (Thung et al., 2016) and 40% in Venezuela (Lopez Gasca et al., 2018). Levofloxacin is a 3^nd^ generation fluoroquinolone currently suggested for *H. pylori* eradication (Malfertheiner et al., 2017); it inhibits DNA gyrase and topoisomerase and mutations in the *gyrA* gene are associated with resistance (Nishizawa and Suzuki, 2014).

Rates of antibiotic resistance may also vary within countries, where differences in drug exposure may be observed within communities. Latin America is a region that conserve a large number of indigenous communities that have remained relatively isolated and often practice a traditional medicine without consumption of drugs. We hypothesized that rates of antibiotic resistance are lower within these communities. In Mexico, approximately 7% of the Mexican population (over 7 million Mexicans) speak a native language. These ethnic groups have different economic development and lifestyles with little access to medical services (Romero-Hidalgo et al., 2017).

Recent technical progress in whole-genome sequencing has allowed the study of drug susceptibility by searching for the known mutations associated with resistance; this approach may led to the discovery of novel mutations responsible for antibiotic resistance. The aim of this work was to compare the susceptibility to antibiotics in *H. pylori* strains isolated in a mestizo community versus isolates from indigenous populations. We also aimed to evaluate the performance of resistance detection by genome sequencing as compared with the phenotypic detection, measuring susceptibility in agar plates.

## Materials and Methods

### Bacterial Strains

A total of 167 *H. pylori* strains isolated during the period from 1997 to 2017 were studied, 111 strains were isolated from biopsies of Mexican mestizo adults with non-atrophic gastritis, intestinal metaplasia, gastric cancer, and duodenal ulcer. Biopsy specimens were cultured as previously described (Avilés-Jiménez et al., 2012). All patients were seen at the Centro Médico Nacional Siglo XXI, Instituto Mexicano del Seguro Social in Mexico City. The study was approved by the ethical committee from the Instituto Mexicano del Seguro Social. In addition, 56 *H. pylori* strains previously isolated from Mexican indigenous people were included (Camorlinga-Ponce et al., 2011). All *H. pylori* organisms were stored at −70°C until tested.

### Phenotypic Characterization of Antimicrobial Susceptibility

The antimicrobial susceptibility of *H. pylori* to amoxicillin, clarithromycin, levofloxacin, and metronidazole was determined using the Epsilometer test (E test) (Torres et al., 2001), a test that whereas is not considered as the reference methods, it is commonly used for a number of antibiotics in different bacteria. A recent study reported a good agreement for levofloxacin, clarithromycin, and metronidazole when compared with the agar dilution method, considered as the gold standard (Miftahussurur et al., 2020). *H. pylori* isolates were grown for 2 days on Columbia blood agar plates (Becton Dickinson, New Jersey, USA) and growth suspended in Columbia broth to achieve a McFarland opacity of 3. Bacterial suspension was spread on Muller Hinton agar medium with 5% of sheep blood (Becton Dickinson, New Jersey, USA) and the E-test strips (Liofilchem, Roseto degli Abruzzi, Italy) were placed on the plate and incubated at 37°C for 72 h with a 10% CO_2_ atmosphere (Nuaire, Plymouth, Minn). The MIC was defined by the point of intersection of the inhibitory zone with the strip. MIC values were defined according to the clinical breakpoints proposed in the sixth version of the EUCAST (European Committee on Antimicrobial Susceptibility Testing, EUCAST, 9^th^ version, 2019) for *H. pylori*. The cutoff values for resistance were >8 mg/L for metronidazole, >0.125 mg/L for amoxicillin, >1 mg/L for levofloxacin, and >0.5 mg/L for clarithromycin. Two reference strains were used as controls, *H. pylori* ATCC 43504 with MICs for clarithromycin, amoxicillin, metronidazole, and levofloxacin of 0.016, 0.016, 64, and 0.064 mg/L, respectively and strain *H. pylori* 26695 with MICs for clarithromycin, amoxicillin, metronidazole, and levofloxacin of 0.98, 0.94, 0.75, and 0.32 mg/L, respectively.

### DNA Extraction and Sequencing

Genomic DNA was extracted from 93 *H. pylori* strains using the DNeasy Mini kit (Qiagen, Hilden, Germany) according to the manufacturer's instructions. Nine isolates were sequenced with the HiSeq 2000 platform (Illumina, San Diego, CA, USA) using the paired-end method, as previously described (Muñoz-Ramírez et al., 2017). The remaining isolates were sequenced at the Weimer laboratory at the University of California, Davis, (USA) within the 100K Pathogen Genome Project (Weimer, 2017). Isolates (65) were sequenced using Illumina HiSeq2500 (Weis et al., 2016; Draper et al., 2017). While 19 were done using Pacific Biosciences as previously described (Kong et al., 2014). PacBio reads were processed with the SMRT Analysis package (version 1.3). Genomes were assembled *de novo* by using the hierarchical genome assembly process (HGAP) and polished using Quiver to obtain final consensus assemblies. For the Illumina paired-end 150 reads, we tested k-mers lengths from 31 to 121 using the VelvetOptimiser script (version 2.2.4) (Zerbino and Birney, 2008). The k-mer length that produced the best assembly according to the N50 contig was used to generate the final assemblies using ABySS (version 2.0.2) (Simpson et al., 2009) (**Supplementary Table 1**).

### Identification of Mutations in Resistance-Associated Genes

Genomic variations were identified in genes conferring antibiotic resistance: 23S rRNA for clarithromycin; *rdxA*, *frxA, rpsU, omp11*, and *dppA* for metronidazole; pbp1, pbp2, pbp3 for amoxicillin; and *gyrA* and *gyrB* for levofloxacin. First, open reading frames (ORFs) were predicted from *de novo* assemblies by a genomic annotation using the Prokka software v1.12 (Seemann, 2014) and the proteome of the 26695 strain (NC_000915) as primary source. Then, genomic coordinates of ORFs were used to extract the entire sequence of target genes. Sequences were then aligned according to genes of the 26695 reference strain (GeneBank accession number U27270.1) using muscle v3.8.31 (Edgar, 2004) and SNPs were called from alignments using SNIPPY (v.4.6.0) (Page et al., 2016) (https://github.com/tseemann/snippy) following the parameters: minimum coverage 20X, a minimum base quality score of 30 and a proportion for variant evidence of 0.9. Mutations were considered according to SNIPPY outputs and visualized using Artemis v18.1.0 (Carver et al., 2012) or MEGAS's sequence editor (Tamura et al., 2013).

### Statistical Analysis

The sociodemographic variables were analyzed as follows, gender and clinical diagnosis were compared with the Kruskal-Wallis test. To compare frequency of resistance to antibiotics between the different periods of isolation the X^2^ linear trend was used. Frequency of resistance to one or more antibiotics among the study populations was analyzed by means of X^2^; ORs (Odds Ratio) and their 95% confidence intervals were also estimated. In all cases, a p-value <0.05 was considered as statistically significant.

## Results

### Characteristics of the *H. pylori* Strains Studied

**Table 1** describes the characteristics of the patients from whom *H. pylori* strains were isolated. A total of 167 *H. pylori* strains were included in this study, of these, 111 were isolated from Mexican mestizos (mean age, 48.7 ± 14.2 years; 34 males and 77 females). The clinical diagnosis in these patients were chronic gastritis, pre-neoplastic lesions, gastric cancer and duodenal ulcer. 56 *H. pylori* strains from Mexican indigenous people were also studied, all individuals with chronic gastritis (mean age 41.63 ± 20.0 years; 21 males and 35 females).

**Table 1:** Characteristics of patients from whom *H. pylori* was isolated in Mexico during 1997–2017.

Time Period	Total	Mestizo population	Native community
	1997–2017	1997–2017	2002–2004
No of strains	n=167	n=111	n=56
*Age			
Age, mean(SD)	50.72 (14.27)	48.7(14.2)	41.63 (20.03)
**Gender			
Male, n(%)	48 (28.7)	34 (30.6)	21 (37.5)
Female, n(%)	119 (71.2)	77 (69.4)	35 (62.5)
Diagnosis			
Gastritis	119	63	56
Pre-neoplastic	18	18	0
Lesions			
Gastric cancer	23	23	0
Duodenal ulcer	7	7	0

*t = - 3.03; p = 0.003.

**X^2^ = 0.795; p = 0.37 OR = 0.74 I. C. 95% = (0.38–1.45).

### Antimicrobial Susceptibility

**Figure 1:**
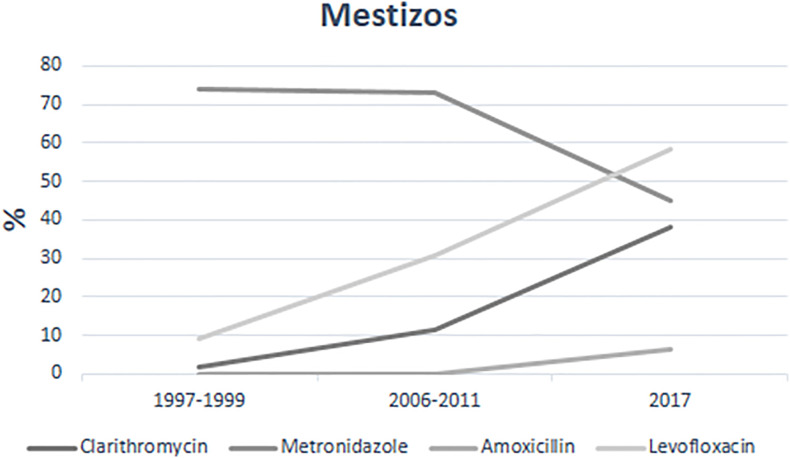
Trends of Antimicrobial resistance in *H. pylori* strains isolated from Mexican mestizos during the period 1997–2017.

Analysis of the antimicrobial susceptibility of the 167 isolates reveled high resistance for metronidazole (58.6%), and moderate to levofloxacin (18.5%) and clarithromycin (8.9%), while only 1.8% were resistant to amoxicillin. We next analyzed the trend of resistance over a 20-year period for the mestizo isolates (**Figure 1** and **Table 2**) and found a constant increase from 1997 to 2017 for clarithromycin (1.85%–32.2%, p=0.000) and levofloxacin (9.2%–58.1%, p= 0.000). In contrast, resistance to metronidazole tend to decrease (73%–51.6%); whereas for amoxicillin resistance was not detected during 1997–2011 but was already present in 6.5% of the 2017 isolates.

**Table 2:** Antimicrobial resistance of *H. pylori* strains isolated from Mexican mestizos over a period of 20 years and from native populations.

Years	^a^Clarithromycin No (%)	^b^Metronidazole No (%)	Amoxicillin No (%)	^c^Levofloxacin No (%)
Mestizo population n=111				
1997–1999(n = 54)	1(1.85)	41(75.9)	0.0	5(9.2)
2006-2011(n=26)	3(11.5)	19(73.0)	0.0	8(30.8)
2017 (n=31)	10(32.2)	16(51.6)	2(6.5)	18(58.1)
Native community				
2002–2004(n=56)	*1 (1.8)	**22 (39.3)	***1 (1.8)	0.0
Total N=167	15(8.9)	98(58.6)	3(1.8)	31(18.5)

^a^X^2^ for linear trend = 15. 87, p = 0.0007; ^b^X^2^ for linear trend = 4.89, p = 0.026; ^c^X^2^ for linear trend = 23.15; p = 0.0000.

*X^2^ = 5.33, p = 0.02, OR = 7.93 (1.01 – 62.0) mestizo vs native populations; **X^2^ = 13.07, p = 0.0002, OR = 3.35 (1.72 – 6.55) mestizo vs native populations; ***Fisher Exact = 0.7, OR = 1.00 (0.89 – 11.37) mestizo vs native populations.

Compared with the mestizo strains, resistance among the isolates of native communities was lower for metronidazole (39.3%), very low for clarithromycin and amoxicillin (1.8% for both) and absent for levofloxacin (0%) (**Table 2**). Among all the 167 *H. pylori* strains tested, 30.5% were susceptible to all four antibiotics tested, which corresponded to 15.3% of the mestizos and 66% of the indigenous communities (**Table 3**). Among all studied strains 56% were resistant to at least one antibiotic and interestingly, 65% of the mestizo vs. 37% of the indigenous strains presented this pattern. Finally, multidrug resistance was also higher in mestizo (18.9%) than in indigenous strains (1.7%) (**Table 3**). Among the multi-resistant mestizo strains, dual resistance to metronidazole/levofloxacin was more frequently observed (16.2%), followed by resistance to clarithromycin/metronidazole/levofloxacin in 4.5% of the tested strains. Resistance to both metronidazole/levofloxacin rose from 7.4% in 1997 to 1999 to 32% in 2017. A high resistance rate of 25.8% was detected for clarithromycin/metronidazole among *H. pylori* isolates from mestizos. No strain was resistant to the four antibiotics tested. Of note, among the strains of indigenous origin, only one isolate presented double resistance (clarithromycin/metronidazole).

**Table 3:** Patterns of antibiotic sensitivity of *H. pylori* strains isolated from indigenous and mestizo communities in Mexico.

Ethnic origin of *H. pylori* strains				
Resistance	Indigenous n=56 (%)	Mestizo n=111 (%)	All n=167 (%)	OR (95% CI)
Sensitive to four antibiotics	34 (66.6)	17 (15.3)	51 (30.5)	13.6 (6.6–27.7)
Resistance to one antibiotic	21 (37.5)	73 (65.7)	94 (56.3)	0.31 (0.16–0.61)
Resistance To > 2 antibiotics	1 (1.7)	21 (18.9)	22 (13.2)	0.07 (0.01–0.59)

OR, Odds Ratio.

### Mutation Analysis in the Resistance Genes

Of the 167 strains included in the study, 93 strains (74 mestizos and 19 indigenous) were whole-genome sequenced, 84 in this study and 9 reported previously (Muñoz-Ramírez et al., 2017) (**Supplementary Table 1**). Seven of these 93 strains were phenotypically resistance to clarithromycin; of these, one presented the A2146G (formerly A2142G) amino acid variant and four presented the variant A2147G (formerly A2143G) (**Table 4**); whereas among the 86 sensitive strains two presented variants reported associated with resistance. In this analysis we checked for mutations in the two copies of the 23S rRNA operon present in *H. pylori*. For levofloxacin, among the 93 strains 22 were resistant and 17 presented amino acid variants in position 87 and 91 of GyrA (**Table 4**); however, 14 of the 71 sensitive also presented these variants. The most common variant found in GyrA was N87I, whereas amino acid variants in GyrB were in two resistant strains but present in four of the 71 sensitive isolates.

**Table 4:** *H. pylori* mutations in genes associated with phenotype resistant to Clarithromycin, Levofloxacin, and Metronidazole.

	Resistant No (%)	Sensitive No (%)
Clarithromycin	n=10	n=100
*23S rRNA*		
A2142G	3(30)	0
A2143G	0	1(1)
A2146G	2(20)	1(1)
A2147G	2(20)	1(1)
Levofloxacin	n=32	n=78
GyrA		
N87T	4(12.5)	7(8.9)
N87K	6(18.8)	3(3.8)
N87I	8(25.0)	5(6.4)
T62I	0	1(1.2)
D91G	1(3.1)	1(1.2)
D91Y	1(3.1)	2(2.5)
N87T-D91G	1(3.1)	0
D91N	1(3.1)	4(5.1)
GyrB		
D481E	0	2(2.5)
D481G	0	1(1.2)
		0
Metronidazole	n=78	n=32
*rpsU*		
A11T	3(3.8)	0
G9S	3(3.8)	0
R36G	0	3(9.3)
*omp11*		
V148I	12(15.3)	12(37.5)
T13A	24(30.7)	31(96.8)

Regarding metronidazole, there are no reports on mutations clearly associated with resistance, accordingly, we describe all the observed mutations. Mutations in *rpsU* leading to variants A11T and G9S were found in resistant strains (**Table 4**); the A11T mutants had a MIC >256 to metronidazole. Two isolates sensitive to metronidazole presented the mutation R36G in *rpsU*. In addition, we detected mutations in *omp11*, but the significance of this finding is unclear because mutations were found in both, strains resistant and sensitive to metronidazole (**Table 4**). In gene *dppA* mutations in I485L, Q382E, and 37del were present only in strains resistant to metronidazole.

A considerable number of mutations was found in *rdxA* and amino acid variants were as much as 64 (**Table 5**), 32 present only in resistant strains, 22 present in both resistant and sensitive and 10 present only in sensitive strains. Besides the 64 non-synonymous mutations in *rdxA* we also found six frameshifts, three nonsense mutations, 32 missense mutations, and stop codons were observed in resistant isolates in positions 13, 26, 37, 72, 73, and 76. Similarly, in FrxA we found no amino acid variants clearly associated with metronidazole resistance, amino acid variants were present only in three resistant *H. pylori* strains, 14 were present in both sensitive and resistant strains and 13 present only in sensitive strains (**Table 6**).

**Table 5:** Amino acid variants present in RdxA of *H. pylori* strains resistant or sensitive to metronidazole.

33 amino acid variants present only in resistant *H. pylori* strains	K2R, Q11X, R14K, H17Y, H25R, E27T, 30SR, A37V, S45N, Q50R, M56V, T58I, L62S, K63Q, S81L, P106D, P106S, V111A, P115R, A118S, C140R,C140G, S158M, G163D, G163V,K168R, A193V, K168R, A193V, E194G, A206T, I207T
10 amino acid variants present only in sensitive *H. pylori* strains	H53R, A69Y, P96L, V123T, S158G, R176H, D205A, A206E, I207E, T208L
22 amino acid variants present in both sensitive and resistant *H. pylori* strains	Q11H, R16G, R16H, T31E, T32A, M56I, D59N, A67V, R90K, H97Y, H97T, G98S, A118T, S128G, R131K, G170S, V172I, E175Q, A183V, C184R, Q197K, V204I.

**Table 6:** Amino acid variants present in FrxA of *H. pylori* strains resistant or sensitive to metronidazole.

3 amino acid variants present only in resistant *H. pylori* strains	L52I, A85V, E169A
13 amino acid variants present only in sensitive *H. pylori* strains	V7I, A16T, A32V, , I44V, R58G, K60R, V81L, D91N, N110D, N124S, A152V, E169A, E176K
14 amino acid variants present in both sensitive and resistant *H. pylori* strains	L33M, A67T, A70G, L71I; F72S, T110S, N110H, F114Y, M125F, N129T, S130D, E176K, N182D, C193S

### Evaluation of the Performance of Genetic Tests to Detect Antibiotic Resistance

To measure the efficacy of molecular detection of resistance, we estimated the concordance of phenotype vs. genotype using the reported antibiotic resistance associated mutations for clarithromycin and levofloxacin and all found mutations for metronidazole (**Table 7**). Performance for clarithromycin had a substantial agreement (K = 0.69), which is as good as in reports from other populations (Farzi et al., 2019; Tshibangu-Kabamba et al., 2020); we found two resistant strains with no mutation in 23S, suggesting that in our population other mechanisms are responsible for this phenotype. Thus, 23S genotyping might be recommended for clarithromycin resistance in *H. pylori* strains from our community. Regarding levofloxacin, genotyping of *gyrA* and *gyrB* had a moderate agreement (*K* = 0.49). Previous studies identified amino acid variants in positions 87 and 91 of GyrA associated with high MIC values for levofloxacin (Lopez Gasca et al., 2018; Hanafiah et al., 2019; Tuan et al., 2019). Genotyping of *gyrA* might not be an alternative to determine levofloxacin resistance if phenotypic tests are not available. For metronidazole, *rdxA* and *frxA* turned out to be highly variable genes with an unusual number of SNPs, and no single mutation clearly associated with resistance resulting in a slight agreement (<0.2); genotyping of *rpsU, dppA*, *rdxA*, or *frxA* is not an option to identify metronidazole resistant strains. Regarding amoxicillin, we found a reduced number of resistant strains and those strains presented no mutations in PBPs. Mutations in resistance-associated genes increased after the period 1997 to 1999 as observed in the periods 2002 to 2011 and 2017 (**Table 8**), particularly for 23S rRNA, *gyrA*, and *dppA* genes.

**Table 7:** Analysis of correlation between antibiotic resistance phenotype and genotype.

Antibiotic	Genotype	Phenotype		Kappa
		S	R	
Clarithromycin	S	97	3	0.67 (0.85–0.91)
	R	3	3	
Levofloxacin	S	53	1	0.5 (0.66–0.71)
	R	27	29	
Metronidazole	S	32	27	0.29 (0.47–0.52)
	R	10	32	

**Table 8:** Mutational analysis in genes involved with antibiotics resistance of Mexican *H. pylori* strains isolated over a period of 20 years.

	1997-1999 n=39	2002-2011 n=40	2017 N=31
*23S rRNA*			
n=7			
A2146G		1(14.2)	3(42.8)
A2147G			1(14.2)
Levofloxacin			
n=22			
* GyrA*			
N87T	0	1(4.5)	0
N87K		1(4.5)	3(13.6)
N87I		2(9)	4(18.1)
D91G			1(4.5)
D91Y			1(4.5)
N87T-D91G		1(4.5)	
D91N		2(9)	1(4.5)
Metronidazole			
*n=59*			
*rpsU*			
A11T	1(1.6)		1(1.6)
G9S		3(5)	
			
*Omp11*			
V148I	5(8.4)	4(6.7)	2(3.3)
T13A	8(13.5)	9(15.2)	3(5)
			
*dppA*			
37del		1(1.6)	3(5)
I485L		4(6.7)	
I485V		3(5)	4(6.7)
A212E		12(20.3)	8(13.5)
Q382E		3(5)	

## Discussion

In 2017, *H. pylori* was categorized by the WHO as a bacterium with high antibiotic resistance that poses an important risk to human health (Dang and Graham, 2017). *H. pylori* antibiotic resistance is the main factor affecting the success of current therapeutic regimens and alternatives for suitable antibiotics to treat this infection are becoming limited. Clarithromycin and amoxicillin are the antibiotics of the standard triple therapy, still in use in many regions for the of eradication *H*. *pylori* (Suzuki et al., 2019). However, the resistance to these antimicrobials has increased rapidly during the last decade in several countries (Ogata et al., 2013; Miftahussurur et al., 2016; Park et al., 2016).

We aimed to study the pattern of antibiotic resistance in Mexican mestizo population over a period of 20 years. During this period, we found a significant increase in the rate of resistance to clarithromycin and levofloxacin and moderate to amoxicillin; while resistance to metronidazole decreased. Resistance to clarithromycin was low in the first year of the survey, but after 20 years, it increased to levels that banned its clinical use; similar findings have been reported in Taiwan (65%) (Wu et al., 2015), Chile (31.2%) (Gonzalez-Hormazabal et al., 2018) and Brazil (19.5%) (Ogata et al., 2013). Based on our studies, the most recent Mexican consensus no longer recommends the standard triple therapy for *H. pylori* eradication (Bosques-Padilla et al., 2018).

Over the 20-year study period, the resistance to metronidazole decreased from 75% to 51.6%, a significant reduction that might be related to the fact that its previous widespread use (to treat diarrhea, parasitic infections or gynecological disorders), which favored the selection of resistant strains, has been drastically reduced in the last decades, particularly after the act that commanded written prescription to get antibiotics (Vázquez Tsuji Óscar, 2010). The current 51% rate of resistance is similar to that reported in Alaska (44%), but lower than China (56.6%), Vietnam (69.9 and %), and Colombia (83%) (Ierardi et al., 2013; Camargo et al., 2015).

After failure of the standard triple therapy, the Maastricht IV guidelines recommended levofloxacin as a salvage therapy (Malfertheiner et al., 2017) and other countries even suggested levofloxacin as first line therapy (Heo and Jeon, 2014; Liou et al., 2016). We found that in Mexico, resistance to levofloxacin rise from 9% to 58% in a 20-year period; a 10% resistance has been suggested as the threshold to use levofloxacin as empiric first line therapy (Thung et al., 2016). Levofloxacin was clinically useful at the beginning of the survey, but now it is useless to eradicate *H. pylori* infection in mestizo population. This work is the first report on levofloxacin resistance in *H. pylori* in Mexico, but several studies have reported high levofloxacin resistance in Europe and Asia (Megraud et al., 2013; Alfizah et al., 2014; Smith et al., 2014; O’Connor et al., 2015). In Mexico a recent clinical trial compared the utility of triple therapy with clarithromycin versus levofloxacin and found a poor efficacy with both treatments with eradication rates close to 60% (Ladrón-de-Guevara et al., 2019). Quinolones have been widely used to treat urinary tract infections and other infections in Mexico, and the observed high resistance in *H. pylori* strains is most probably the result of exposure when other infections are treated. Studies reporting antibiotic resistance over a period of years are scarce in Latin America, our work clearly show a sharp increase in resistance to clarithromycin and levofloxacin and also the worrying appearance of resistance to amoxicillin. These pronounced changes in susceptibility are an indication of the need to monitor patterns of resistance, particularly in countries with high prevalence of the infection, including the Latin American region (Curado et al., 2019). The resistance rates to clarithromycin, metronidazole, and levofloxacin have increased over time globally, which agrees with studies presenting evidence of an association between antibiotic consumption and antibiotic resistant in *H. pylori* infection (Megraud et al., 2013; Camargo et al., 2015). In Mexico clarithromycin, amoxicillin and metronidazole are antibiotics commonly used to treat other infections, which would also favor this increase in resistance. Therapies including levofloxacin are currently recommended; however, in *H. pylori* isolates from 2017, we found levofloxacin resistance in 58% and multiresistance to metronidazole and levofloxacin of 32%, discarding their clinical use for this infection.

A second aim of this study was to contrast the antimicrobial susceptibility to the most commonly used antibiotics in Mexican *H. pylori* strains isolated from two ethnically different groups. We found that *H. pylori* strains from mestizo population presented higher drug resistance than isolates from native communities. Native communities in Mexico usually follow its cultural customs including traditional medicine where the use of antibiotics is limited (Guzman-Rosas, 2016). It is probably the limited exposure to these drugs the cause of reduced antimicrobial resistance, a positive correlation that not always is observed. There are no reports on the treatment to eliminate *H. pylori* infection in native Mexican people. Our hypothesis was further strengthened with the observation of absence of resistance to levofloxacin an antibiotic that hardly reach these communities. As mentioned above, the most recent Mexican consensus no longer recommend triple therapy (Bosques-Padilla et al., 2018) based on susceptibility patterns in mestizo populations. Our results suggest triple therapy may still be an effective treatment in native communities and call the attention to differences between populations that must be taken into account when working in consensus for regions with both mestizo and native communities. This is regionally important since most Latin American countries still have indigenous populations (Adhikari et al., 2016).

Bacteria use two major genetic strategies to adapt to the exposure of antibiotics. One is mutations in genes associated with mechanism of action of the drugs and the second is incorporation of resistance gene by acquisition of foreign DNA via horizontal transfer (Munita and Arias, 2016; Tuan et al., 2019). Several reports have identified mutations in the genome of *H. pylori* involved in resistance to clarithromycin, fluoroquinolones, and metronidazole. Sequencing of the *H. pylori* genome is increasingly more available and less expensive and offers a unique opportunity to identify known and novel mutations in genes associated with resistance to antibiotics. Experimentally confirmed mutations can then be identified using even simpler and cheaper techniques, some commercially available and designed for use with biopsy tissues without the need to isolate *H. pylori* (Poel et al., 2019; Pichon et al., 2020). However, this is only possible after a thorough confirmation of the specific association with resistance in different regions of the world.

*H. pylori* resistance to clarithromycin is frequently due to point mutation in the 23s rRNA gene and prevalence of these mutations varies geographically even between populations in the same country (Kageyama et al., 2019). In Chinese patients, prevalence of A2143G was 10%–14% (Savoldi et al., 2018; Zhuoqi et al., 2008) and in Malaysian strains it was 90.5% (Alfizah et al., 2014). Furthermore, in Iran A2143G was highly frequent in clarithromycin resistant strains (68.7%) and A2142G was low (5.6%) (Keshavarz et al., 2015); similarly, A2142G was low (9.5%) in *H. pylori* resistant strains from Malaysia (Alfizah et al., 2014), whereas in Brazil A2147G variant was the most common (77.8%) (Martins et al., 2006). We found good correlation between phenotypic resistant to clarithromycin and mutations in 23s rRNA, mainly because the reported mutations were present in resistant strains and mostly absent in sensitive strains. There were however strains resistant to clarithromycin but without mutations in 23s rRNA suggesting the presence of other mechanism of resistance in *H. pylori* in our population.

Resistance to levofloxacin is given by non-synonymous mutations in a region of *gyrA* or gyrB, determining resistance to quinolones. Amino acid substitutions in GyrA have been described in positions 91 (D91G, N, A, Y, or H) and 87 (N87L, I, A, or K) (Zerbetto De Palma et al., 2017) in addition to other substitutions in GyrB. 87 and 91 substitutions are the more frequently described, suggesting their presence might predict resistance to fluoroquinolones (Zerbetto De Palma et al., 2017; Lopez Gasca et al., 2018). In Colombia an important increase in fluoroquinolone resistance from 2009 to 2014 was reported, mostly associated with mutations in *gyrA*, with N87I being the most common variant (Wang et al., 2010; Trespalacios et al., 2015). In our study, we did not find any substitution in GyrA exclusively present in resistant strains and in fact the performance of molecular detection of mutants was moderate in our population, mainly because of the frequent presence of the described resistant-associated variants in sensitive strains. Limiting the test to the detection of variants in position 87 of GyrA increase concordance but sensitivity is still low (detecting in 77% of resistant strains). *gyrB* mutation (S479G) was identified in two *H. pylori* resistant strains, indicating that *gyr B* has no impact on fluoroquinolone resistance in our population. Our results agree with previous reports from Kuala Lumpur and Iran (Hanafiah et al., 2019; Tuan et al., 2019).

Resistance to metronidazole involves the inactivation of the *rdxA* gene that catalyzes the reduction of metronidazole (Tuan et al., 2019). A number of studies have reported amino acid variants in RdxA present only in metronidazole resistant strains (Marais et al., 2003; Butlop et al., 2016). In this study we found amino acid variants in RdxA that were associated exclusively with metronidazole resistant strains in over 50% of the resistant isolates; however, these variants were as much as 32 and none was present in more than 5% of resistant isolates. In addition, 32 other variants were present either in both sensitive and resistant strains or only in sensitive isolates. Thus, it is not a surprise the low concordance found with molecular and phenotypic detection; in addition, considering the large number on mutations in the *rdxA* gene it seems that molecular detection of resistance is not an option for metronidazole. Furthermore, six metronidazole-resistant strains contained stop codons at position 13, 26, 37, 72, 73, 73; different from those reported previously in positions 2, 50, and 52 (Butlop et al., 2016). We found a number of amino acid variants present only in resistant *H. pylori* isolates. Sustitutions in *rpsU* (A11T) and *dppA* (I485L, 37del) were found to be present only in resistant isolates however, the role of these variants in resistance to metronidazole remains to be elucidated because we had a high percentage of resistant strains that did not present any of the studied mutations, whereas other mutations were present in both sensitive and resistant strains. The comparison between the phenotypic and genotypic methods is reported for different authors in various countries and with diverse results (De Francesco et al., 2010; Tuan et al., 2019; Mascellino et al., 2020). Mascellino et al, reported good correlation genotype-phenotype among *H. pylori* resistant to levofloxacin but not in clarithromycin resistant strains (Mascellino et al., 2020). While Tuan et al. (2019) reported excellent genotype to phenotype agreement for clarithromycin and good agreement for levofloxacin and amoxicillin but no agreement for metronidazole.

We have to keep in mind that other mechanisms of metronidazole resistance have been described, including mutations in frxA, *dapF*, and efflux proteins (Miftahussurur et al., 2016) (**Table 8**).

In this study, we showed that in Mexican *H. pylori* strains there was an important increase in resistance to clarithromycin and levofloxacin and appearance of resistant to amoxicillin during a period of 20 years, which has important clinical implications. We also report a significantly lower frequency of antibiotics resistance in *H. pylori* strains from native communities. Finally, we found good to moderate performance of genetic test to detect antibiotic resistance; thus, molecular methods for characterizing resistant genes require further validation in each population.

## Data Availability Statement

The data set analyzed in this work is publicly accessible under the NCI bioproject PRJNA338771 (biosamples: SAMN05569559, SAMN05569561, SAMN05569574, SAMN05569575, SAMN05569581-SAMN05569583, SAMN05569586, SAMN05569587, SAMN05569589-SAMN05569592, SAMN11483334 and SAMN11483336); bioproject PRJNA203445 (biosamples: SAMN09935045-SAMN09935055, SAMN09935057-SAMN09935061, SAMN09935063, SAMN09935064, SAMN10881935, SAMN10881940, SAMN10881942, SAMN10881945-SAMN10881948, SAMN10881953, SAMN10881959, SAMN10881963, SAMN10881965, SAMN10881972) and bioproject PRJNA681870 (biosamples SAMN16970063-SAMN16970092 and SAMN16970108-SAMN16970125) (**Supplementary Table 1**).

## Ethics Statement

The study was approved by the ethical committee from the Instituto Mexicano del Seguro Social.

## Author Contributions

MC-P designed and coordinated the study and wrote the manuscript. JT participated in design and coordination of the study, and wrote the manuscript. AE-O performed the isolation and antimicrobial susceptibility test of *H. pylori*. AG-D participated in statistical analysis. EA-Z and RT participated in the bioinformatics analysis. SG-C contributed with revising the article. All authors contributed to the article and approved the submitted version.

## Funding

This work was supported by “Coordinación Nacional de Investigación en Salud, Instituto Mexicano del Seguro Social. México. Grand FIS/IMSS/PROT/G16/1600.

## Conflict of Interest

The authors declare that the research was conducted in the absence of any commercial or financial relationships that could be construed as a potential conflict of interest.

## References

[B1] Adhikari K. Mendoza-Revilla J. Chacón-Duque J. C. Fuentes-Guajardo M. Ruiz-Linares A. (2016). Admixture in Latin America. Curr. Opin. Genet. Dev. 41, 106–114. 10.1016/j.gde.2016.09.003 27690355

[B2] Alarcón-Millán J. Fernández-Tilapa G. Cortés-Malagón E. M. Castañón-Sánchez C. A. De Sampedro-Reyes J. Cruz-del Carmen I. (2016). Clarithromycin resistance and prevalence of *Helicobacter pylori* virulent genotypes in patients from Southern México with chronic gastritis. Infect. Genet. Evol. 44, 190–198. 10.1016/j.meegid.2016.06.044 27355861

[B3] Alba C. Blanco A. Alarcón T. (2017). Antibiotic resistance in *Helicobacter pylori*. Curr. Opin. Infect. Dis. 30, 489–497. 10.1097/QCO.0000000000000396 28704226

[B4] Alfizah H. Norazah A. Hamizah R. Ramelah M. (2014). Resistotype of *Helicobacter pylori* isolates: The impact on eradication outcome. J. Med. Microbiol. 63, 703–709. 10.1099/jmm.0.069781-0 24757218

[B5] Avilés-Jiménez F. Reyes-Leon A. Nieto-Patlán E. Hansen L. Burgueño J. Ramos I. (2012). In Vivo Expression of *Helicobacter pylori* Virulence Genes in Patients With Gastritis, Ulcer, and Gastric Cancer. Infect. Immun. 80, 594–601. 10.1128/IAI.05845-11 22124657 PMC3264312

[B6] Bosques-Padilla F. J. Remes-Troche J. M. González-Huezo M. S. Pérez-Pérez G. Torres-López J. Abdo-Francis J. M. (2018). IV consenso mexicano sobre *Helicobacter pylori*. Rev. Gastroenterol. México 83, 325–341. 10.1016/j.rgmx.2018.05.003 29941237

[B7] Butlop T. R. P. Mungkote N. T. N. Chaichanawongsaroj N. T. R. (2016). Analysis of allelic variants of rdxA associated with metronidazole resistance in *Helicobacter pylori*: detection of common genotypes in rdxA by multiplex allele-specific polymerase chain reaction. Genet. Mol. Res. 15, 1–11. 10.4238/gmr.15038674 27706703

[B8] Camargo M. C. García A. Riquelme A. Camargo C. A. Hernandez-garcía T. Candia R. (2015). The problem of Helicobacter pylori resistance to antibiotics: a systematic Review in Latin America. Am. J. Gastroenterol. 2014, 485–495. 10.1038/ajg.2014.24.The PMC4268863 24589670

[B9] Camorlinga-Ponce M. Perez-Perez G. Gonzalez-Valencia G. Mendoza I. Peñaloza-Espinosa R. Ramos I. (2011). Helicobacter pylori genotyping from american indigenous groups shows novel amerindian vacA and cagA alleles and Asian, African and European admixture. PloS One 6 (11), e 27212. 10.1371/journal.pone.0027212 PMC3207844 22073291

[B10] Carver T. Harris S. R. Berriman M. Parkhill J. McQuillan J. A. (2012). Artemis: An integrated platform for visualization and analysis of high-throughput sequence-based experimental data. Bioinformatics 28, 464–469. 10.1093/bioinformatics/btr703 22199388 PMC3278759

[B11] Curado M. Moura de Oliveira M. Araujo M. (2019). Prevalence of *Helicobacter Pylori* Infection in Latin America and the Caribbean Populations: A Systematic Review and Meta-Analysis. Cancer Epidemiol. 60, 141–148. 10.1016/j.canep.2019.04.003 31009922

[B12] Dadgostar P. (2019). Antimicrobial resistance: implications and costs. Infect. Drug Resist. 12, 3903–3910. 10.2147/IDR.S234610 31908502 PMC6929930

[B13] Dang B. Graham D. (2017). *Helicobacter pylori* Infection and Antibiotic Resistance: A WHO High Priority? Nat. Rev. Gastroenterol. Hepatol. 14, 383–384. 10.1038/nrgastro.2017.57 28465548 PMC6905073

[B14] De Francesco V. Zullo A. Ierardi E. Giorgio F. Perna F. Hassan C. (2010). Phenotipic and genotypic *Helicobacter pylori* clarithromycin resistance and therapeutic outcome: benefits and limits. J. Antimicrob. Chemother. 65 (2), 327–332. 10.1093/jac/dkp445 20008044

[B15] Draper J. L. Hansen L. M. Bernick D. Abedrabbo S. Underwood J. G. Kong N. (2017). Fallacy of the Unique Genome: Sequence Diversity within Single Helicobacter pylori Strains. mBio 8 (1), e02321–16. 10.1128/mBio.02321-16 PMC5358919 28223462

[B16] Edgar R. C. (2004). MUSCLE: Multiple sequence alignment with high accuracy and high throughput. Nucleic Acids Res. 32, 1792–1797. 10.1093/nar/gkh340 15034147 PMC390337

[B17] Farzi N. Yadegar A. Sadedhi A. Asadzadeh A. Marian S. Raymond J. (2019). High Prevalence of Antibiotic Resistance in Iranian *Helicobacter pylori* Isolates: Importance of Functional and Mutational Analysis of Resistance Genes and Virulence Genotyping. J. Clin. Med. 8 (11), 1–18. 10.3390/jcm8112004 PMC6912791 31744181

[B18] Fiorini G. Zullo A. Saracino I. M. Gatta L. Pavoni M. Vaira D. (2018). Pylera and sequential therapy for first-line *Helicobacter pylori* eradication. Eur. J. Gastroenterol. Hepatol. 30, 621–625. 10.1097/MEG.0000000000001102 29481383

[B19] Fuccio L. Henry Eusebi L. Maurizio Zagari R. Bazzoli F. (2009). *Helicobacter pylori* Eradication Treatment Reduces but Does Not Abolish the Risk of Gastric Cancer. Am. J. Gastroenterol. 104, 3100–3101. 10.1038/ajg.2009.516 19956126

[B20] Goderska K. Agudo Pena S. Alarcon T. (2018). *Helicobacter pylori* treatment: antibiotics or probiotics. Appl. Microbiol. Biotechnol. 102, 1–7. 10.1007/s00253-017-8535-7 29075827 PMC5748437

[B21] Gonzalez-Hormazabal P. Musleh M. Escandar S. Valladares H. Lanzarini E. Castro V. (2018). Prevalence of Clarithromycin Resistance in *Helicobacter pylori* in Santiago, Chile, Estimated by Real-Time PCR Directly From Gastric Mucosa. BMC. Gastroenterol. 18, 91–91. 10.1186/s12876-018-0820-0 29925321 PMC6011593

[B22] Goodwin A. Kersulyte D. Sisson G. Veldhuyzen van Zanten S. J. Berg D. E. Hoffman P. S. (1998). Metronidazole resistance in *Helicobacter pylori* is due to null mutations in a gene (rdxA) that encodes an oxygen-insensitive NADPH nitroreductase. Mol. Microbiol. 28, 383–393. 10.1046/j.1365-2958.1998.00806.x 9622362

[B23] Guzman-Rosas S. (2016). La interculturalidad en salud: espacio de convergencia entre dos sistemas de conocimiento*. Rev. Gerenc. Polít. Salud. 15, 10–29. 10.11144/Javeriana.rgyps15-31.isec

[B24] Hanafiah A. Binmaeil H. Raja R. Mohamed I. Lopes B. (2019). Molecular Characterization and Prevalence of Antibiotic Resistance in *Helicobacter pylori* Isolates in Kuala Lumpur, Malaysia. Infect. Drug Resistant 12, 3051–3061. 10.2147/IDR.S219069 PMC6774992 31632095

[B25] Hashemi S. Farajzadeh A. Goodarzi H. Jaafar M. Saeid S. Aslani S. (2019). Genetic basis for metronidazole and clarithromycin resistance in *Helicobacter pylori* strains isolated from patients with gastroduodenal disorders. Infect. Drug Resistant 12, 535–543. 10.3390/jcm9061930 PMC6404679 30881059

[B26] Heo J. Jeon S. W. (2014). Optimal treatment strategy for *Helicobacter pylori*: era of antibiotic resistance. World J. Gastroenterol. 20, 5654–5659. 10.3748/wjg.v20.i19.5654 24914324 PMC4024773

[B27] Hooi J. Ying W. Khoon W. Suen M. Underwood F. Tanyingoh D. (2017). Global Prevalence of *Helicobacter pylori* Infection: Systematic Review and Meta-Analysis. Gastroenterology 153, 420–429. 10.1053/j.gastro.2017.04.022 28456631

[B28] Ierardi E. Giorgio F. Losurdo G. Di Leo A. Principi M. (2013). How antibiotic resistances could change *Helicobacter pylori* treatment: A matter of geography? World J. Gastroenterol. 19, 8168–8180. 10.3748/wjg.v19.i45.8168 24363506 PMC3857438

[B29] Kageyama C. Sato M. Sakae H. Obayashi Y. Kawahara Y. Mima T. (2019). Increase in antibiotic resistant *Helicobacter pylori* in a University Hospital in Japan. Infect. Drug Resist. 12, 597–602. 10.2147/IDR.S196452 30881065 PMC6419596

[B30] Keshavarz A. Moniri R. Saffari M. Razavi Zadeh M. Arj A. Mousavi S. G. A. (2015). The *Helicobacter pylori* Resistance Rate to Clarithromycin in Iran. Microb. Drug Resist. 21, 69–73. 10.1089/mdr.2014.0104 25144338

[B31] Kong N. Thao K. Ng W. Agulto R. Weiss A. Spittle K. (2014). Automation of PacBio SMRTbell 10 kb Template Preparation on an Agilent NGS Workstation. Agil. Technol. 10.13140/RG.2.1.4403.2725

[B32] Ladrón-de-Guevara L. Bornstein-Quevedo L. González-Huezo S. Castañeda-Romero B. Costa F. G. di Silvio-López M. (2019). *Helicobacter pylori* eradication in Mexico with a levofloxacin-based scheme versus standard triple therapy: Results from an open-label, randomized, noninferiority phase iiib trial. Rev. Gastroenterol. Mex. 84, 274–283. 10.1016/j.rgmx.2018.04.005 30060902

[B33] Laxminarayan R. Duse A. Wattal C. Zaidi A. K. M. Wertheim H. F. L. Sumpradit N. (2013). Antibiotic resistance — the need for global solutions. Lancet Infect. Dis. 13, 1057–1098. 10.1016/S1473-3099(13)70318-9 24252483

[B34] Leja M. Axon A. Brenner H. (2016). Epidemiology of *Helicobacter pylori* infection. Helicobacter 21, 3–7. 10.1111/hel.12332 27531531

[B35] Liou J.-M. Bair M.-J. Chen C.-C. Lee Y.-C. Chen M.-J. Chen C.-C. (2016). Levofloxacin Sequential Therapy vs Levofloxacin Triple Therapy in the Second-Line Treatment of *Helicobacter pylori*: A Randomized Trial. Am. J. Gastroenterol. 111, 381–387. 10.1038/ajg.2015.439 26832653

[B36] Lopez Gasca M. Peña J. Garcia Amado M. Michelangeli F. Contreras M. (2018). Point Mutations at gyrA and gyrB Genes of Levofloxacin-Resistant *Helicobacter pylori* Isolates in the Esophageal Mucosa From a Venezuelan Population. Am. J. Trop. Med. Hyg. 98, 1051–1055. 10.4269/ajtmh.17-0478 29405113 PMC5928819

[B37] Malfertheiner P. Megraud F. O’Morain C. A. Gisbert J. P. Kuipers E. J. Axon A. T. (2017). Management of *Helicobacter pylori* infection-the Maastricht V/Florence consensus report. Gut 66, 6–30. 10.1136/gutjnl-2016-312288 27707777

[B38] Marais A. Bilardi C. Cantet F. Mendz G. L. Mégraud F. (2003). Characterization of the genes rdxA and frxA involved in metronidazole resistance in *Helicobacter pylori*. Res. Microbiol. 154, 137–144. 10.1016/S0923-2508(03)00030-5 12648728

[B39] Marques A. T. Vitor J. Santos A. Oleastro M. Vale F. (2020). Trends in *Helicobacter pylori* resistance to clarithomycin: from phenotypic to genomic approaches. Microbial. Genomics 6, 1–11. 10.1099/mgen.0.000344 PMC7200067 32118532

[B40] Martins L. C. de Oliveira Corvelo T. C. Oti H. T. do Socorro Pompeu Loiola R. Aguiar D. C. F. dos Santos Barile K. A. (2006). ABH and Lewis antigen distributions in blood, saliva and gastric and *H pylori* infection in gastric ulcer patients. World J. Gastroenterol. 12, 1120–1124. 10.3748/wjg.v12.i7.1120 16534856 PMC4087907

[B41] Mascellino M. Oliva A. Miele M. De Angelis M. Bruno G. (2020). Secondary antibiotic resistance, correlation gentypic and phenotypic methods and treatment in *Helicobacter pylori* infected patients: A retrospective study. Antibiotics 9, 549–559. 10.3390/antibiotics9090549 PMC7560230 32872117

[B42] Megraud F. Coenen S. Versporten A. Kist M. Lopez-Brea M. Hirschl A. M. (2013). *Helicobacter pylori* resistance to antibiotics in Europe and its relationship to antibiotic consumption. Gut 62, 34–42. 10.1136/gutjnl-2012-302254 22580412

[B43] Mera R. Fontham E. T. H. Bravo L. E. Bravo J. C. Piazuelo M. B. Camargo M. C. (2005). Long term follow up of patients treated for *Helicobacter pylori* infection. Gut 54, 1536–1540. 10.1136/gut.2005.072009 15985559 PMC1462952

[B44] Miftahussurur M. Syam A. F. Nusi I. A. Makmun D. Waskito L. A. Zein L. H. (2016). Surveillance of *Helicobacter pylori* antibiotic susceptibility in Indonesia: Different resistance types among regions and with novel genetic mutations. PloS One 11, 1–17. 10.1371/journal.pone.0166199 PMC5131997 27906990

[B45] Miftahussurur M. Fauzia K. A. Nusi I. A. Setiawan P. B. Syam A. F. Waskito L. A. (2020). E-test versus agar dilution for antibiotic susceptibility testing of *Helicobacter* pylori: a comparison study. BMC Res. Notes 13, 9. 10.1186/s13104-019-4877-9 31924273 PMC6954499

[B46] Moodley Y. Linz B. Bond R. P. Nieuwoudt M. Soodyall H. Schlebusch C. M. (2012). Age of the association between *Helicobacter pylori* and man. PloS Pathog. 8, e1002693. 10.1371/journal.ppat.1002693 22589724 PMC3349757

[B47] Moss S. F. (2017). The Clinical Evidence Linking Helicobacter pylori to Gastric Cancer. Cell Mol. Gastroenterol. Hepatol. 3, 183–191. 10.1016/j.jcmgh.2016.12.001 28275685 PMC5331857

[B48] Munita J. M. Arias C. A. (2016). Mechanisms of Antibiotic Resistance. Microbiol. Spectr. 4, 1–37. 10.1128/microbiolspec.VMBF-0016-2015 PMC4888801 27227291

[B49] Muñoz-Ramírez Z. Y. Mendez-Tenorio A. Kato I. Bravo M. M. Rizzato C. Thorell K. (2017). Whole genome sequence and phylogenetic analysis show *Helicobacter pylori* strains from Latin America have followed a unique evolution pathway. Front. Cell. Infect. Microbiol. 7, 50. 10.3389/fcimb.2017.00050 28293542 PMC5328995

[B50] Nishizawa T. Suzuki H. (2014). Mechanisms of *Helicobacter pylori* antibiotic resistance and molecular testing. Front. Mol. Biosci. 1, 19. 10.3389/fmolb.2014.00019 25988160 PMC4428472

[B51] Ogata S. K. Godoy A. P. O. da Silva Patricio F. R. Kawakami E. (2013). High *Helicobacter pylori* resistance to metronidazole and clarithromycin in Brazilian children and adolescents. J. Pediatr. Gastroenterol. Nutr. 56, 645–648. 10.1097/MPG.0b013e31828b3669 23403439

[B52] O’Connor A. Gisbert J. P. O’Morain C. Ladas S. (2015). Treatment of *Helicobacter pylori* Infection 2015. Helicobacter 20 Suppl 1, 54–61. 10.1111/hel.12258 26372826

[B53] Page A. J. Taylor B. Delaney A. J. Soares J. Seemann T. Keane J. A. (2016). SNP-sites: rapid efficient extraction of SNPs from multi-FASTA alignments. Microb. Genomics 2 1-5 (4), e000056. 10.1099/mgen.0.000056 PMC5320690 28348851

[B54] Park J. Y. Dunbar K. B. Mitui M. Arnold C. A. Lam-Himlin D. M. Valasek M. A. (2016). *Helicobacter pylori* Clarithromycin Resistance and Treatment Failure Are Common in the USA. Dig. Dis. Sci. 61, 2373–2380. 10.1007/s10620-016-4091-8 26923948

[B55] Pichon M. Pichard B. Barrioz T. Plouzenau C. Croquet V. Fotsing G. (2020). Diagnostic Accuracy of a Non-Invasive Test for the Detection of *Helicobacter pylori and* Resistance to Clarithromycin in Stool by Real-Time PCR Amplidiag ® H. pylori + ClariR Assay. J. Clin. Microbiol. 58 (4), e01787–19. 10.1128/JCM.01787-19 PMC7098740 31996442

[B56] Poel B. Gils S. Micalessi I. Carton S. Christiaens P. Cuyle P. J. (2019). Molecular Detection of *Helicobacter pylori and* Clarithromycin Resistance in Gastric Biopsies: A Prospective Evaluation of RIDA®GENE Helicobacter pylori Assay. Acta Clin. Belg. 17, 1–7. 10.1080/17843286.2019.1685741 31662122

[B57] Rimbara E. Noguchi N. Kijima H. Yamaguchi T. Kawai T. Sasatsu M (2007). Mutations in the 23S rRNA gene of clrarithromycin resistant *Helicobacter pylori* from Japan. Int. J. Antimicro Agents 30 (3), 250–254. 10.1016/j.ijantimicag.2007.04.009 17590317

[B58] Rollan A. Cortés P. Durán L. Pablo Arab J. Candia R. Espino A. (2014). Clinical trials study Management of *Helicobacter pylori* infection in Latin America: A Delphi technique-based consensus. World J. Gastroenterol. 20, 10969–10983. 10.3748/wjg.v20.i31.10969 25152601 PMC4138478

[B59] Romero-Hidalgo S. Ochoa-Leyva A. Garcíarrubio A. Acuña-Alonzo V. Antúnez-Argüelles E. Balcazar-Quintero M. (2017). Demographic history and biologically relevant genetic variation of Native Mexicans inferred from whole-genome sequencing. Nat. Commun. 8, 1005. 10.1038/s41467-017-01194-z 29044207 PMC5647344

[B60] Saranathan R. Levi M. Wattan A. Malek A. Asare E. Behin D. (2020). *Helicobacter pylori* infectious in the Bronx, New York: Surveying antibiotic susceptibility and strain lineage by whole-genome sequencing. J. Clin. Microbiol. 58 (3), 1–13. 10.1128/JCM.01591-19 PMC7041580 31801839

[B61] Savoldi A. Carrara E. Graham D. Y. Conti M. Tacconelli E. (2018). Prevalence of antibiotic resistance in *Helicobacter pylori*: A systematic Review and metaanalysis in World Health Organization Regions. Gastroenterology 155 (5), 1372–1382. 10.1053/j.gastro.2018.07.007 29990487 PMC6905086

[B62] Seemann T. (2014). Prokka: rapid prokaryotic genome annotation. Bioinformatics 30, 2068–2069. 10.1093/bioinformatics/btu153 24642063

[B63] Sheu B.-S. Wu M.-S. Chiu C.-T. Lo J.-C. Wu D.-C. Liou J.-M. (2017). Consensus on the clinical management, screening-to-treat, and surveillance of *Helicobacter pylori* infection to improve gastric cancer control on a nationwide scale. Helicobacter 22, 1–15. 10.1111/hel.12368 PMC5434958 28066960

[B64] Simpson J. T. Wong K. Jackman S. D. Shein J. E. Jones S. J. Birol l (2009). ABySS: A parallel assembler for short read sequence data ABySS : A parallel assembler for short read sequence data. Genome Res. 19, 1117–1123. 10.1101/gr.089532.108 PMC2694472 19251739

[B65] Sjomina O. Pavlova J. Niv Y. Leja M. (2018). Epidemiology of *Helicobacter pylori* infection. Helicobacter 23 suppl 1, 1–15. e12514. 10.1111/hel.12514 30203587

[B66] Smith S. M. O’Morain C. McNamara D. (2014). Antimicrobial susceptibility testing for *Helicobacter pylori* in times of increasing antibiotic resistance. World J. Gastroenterol. 20, 9912–9921. 10.3748/wjg.v20.i29.9912 25110421 PMC4123372

[B67] Suzuki S. Esaki M. Kusano C. Ikehara H. Gotoda T. (2019). Development of *Helicobacter pylori* treatment: How do we manage antimicrobial resistance? World J. Gastroenterol. 25, 1907–1912. 10.3748/wjg.v25.i16.1907 31086459 PMC6487377

[B68] Tamura K. Stecher G. Peterson D. Filipski A. Kumar S. (2013). MEGA6: Molecular Evolutionary Genetics Analysis version 6.0. Mol. Biol. Evol. 30, 2725–2729. 10.1093/molbev/mst197 24132122 PMC3840312

[B69] Thung I. Aramin H. Vavinskaya V. Gupta S. Park J. Y. Crowe S. E. (2016). Review article: the global emergence of *Helicobacter pylori* antibiotic resistance. Aliment. Pharmacol. Ther. 43, 514–533. 10.1111/apt.13497 26694080 PMC5064663

[B70] Torres J. Camorlinga-Ponce M. Pérez-Përez G. Madrazo-De la Garza A. Dehesa M. González-Valencia G. (2001). Increasing multidrug resistance in Helicobacter pylori strains isolated from children and adults in Mexico. J. Clin. Microbiol. 39, 2677–2680. 10.1128/JCM.39.7.2677-2680.2001 11427594 PMC88210

[B71] Trespalacios A. A. Rimbara E. Otero W. Reddy R. Graham D. Y. (2015). Improved allele-specific PCR assays for detection of clarithromycin and fluoroquinolone resistant of *Helicobacter pylori* in gastric biopsies: identification of N87I mutation in GyrA. Diagn. Microbiol. Infect. Dis. 81, 251–255. 10.1016/j.diagmicrobio.2014.12.003 25600075 PMC6905078

[B72] Tshibangu-Kabamba E. De jesus P. Phuoc V. Matzumoto T. Akada J. Kido Y. (2020). Next generation sequencing of the whole bacterial genome for tracking molecular insight into the broad spectrum antimicrobial resistance of Helicobacter pylori clinical isolates from the Democractic Republic of Congo. Microorganisms 8, 1–16. 10.3390/microorganisms8060887 PMC7356661 32545318

[B73] Tuan V. P. Narith D. Tshibangu-kabamba E. Dang H. Dung Q. (2019). A Next-Generation Sequencing-Based Approach to Identify Genetic Determinants of Antibiotic Resistance in Cambodian *Helicobacter pylori* Clinical Isolates J. Clin. Med. 8 (6), 858. 10.3390/jcm8060858 PMC6617194 31208076

[B74] Vázquez Tsuji Óscar C. R. T. (2010). Regulación de la venta de antibióticos en México. Rev. Enfer. Infect. Pediatr. XXIII, 47.

[B75] Wang L. Cheng H. Hu F. Li J. (2010). Distribution of gyrA mutations in fluoroquinolone-resistant *Helicobacter pylori* strains World J. Gastroenterol. 16, 2272–2277. 10.3748/wjg.v16.i18.2272 20458765 PMC2868221

[B76] Weimer B. C. (2017). 100K Pathogen Genome Project. Genome Announc. 5 (28), 3–4. 10.1128/genomea.00594-17 PMC5511910 28705971

[B77] Weis A. M. Storey D. B. Taff C. C. Townsend A. K. Huang B. C. Kong N. T. (2016). Genomic Comparison of Campylobacter spp. and Their Potential for Zoonotic Transmission between Birds, Primates, and Livestock. Appl. Environ. Microbiol. 82 (24), 7165–7175. 10.1128/aem.01746-16 27736787 PMC5118927

[B78] Wu T. Chuah S. Lee C. H. Liang C. M. Lu L. Kuo Y. H. (2015). Five-year Sequential Changes in Secondary Antibiotic Resistance of *Helicobacter pylori* in Taiwan. World J. Gastroenterol. 21, 106669–106674. 10.3748/wjg.v21.i37.10669 PMC4588089 26457027

[B79] Zerbetto De Palma G. Mendiondo G. Wonaga A. Viola L. (2017). Occurrence of Mutations in the Antimicrobial Target Genes Related to Levofloxacin, Clarithromycin, and Amoxicillin Resistance in *Helicobacter pylori* Isolates from Buenos Aires C. Microb. Drug Resist. 23, 351–358. 10.1089/mdr.2015.0361 27391421 PMC5397209

[B80] Zerbino D. R. Birney E. (2008). Velvet: Algorithms for de novo short read assembly using de Bruijn graphs. Genome Res. 18 (5), 821–882. 10.1101/gr.074492.107 18349386 PMC2336801

[B81] Zhuoqi L. Shen J. Zhang L. Shen L. Baozhen Q. Zhou J. (2008). Prevalence of A2143G mutation of *H. pylori*-23S rRNA in Chinese subjects with and without clarithromycin use history. BMC Microbiol. 8, 1–8. 10.1186/1471-2180-8-81 18507832 PMC2427034

